# Organ-level distribution tandem mass spectrometry analysis of three structural types of brassinosteroids in rapeseed

**DOI:** 10.3389/fpls.2024.1308781

**Published:** 2024-03-07

**Authors:** Jianhua Tong, Wenkui Zhao, Keming Wang, Danyi Deng, Langtao Xiao

**Affiliations:** ^1^ Hunan Provincial Key Laboratory of Phytohormones and Growth Development, Laboratory of Yuelu Mountain, College of Bioscience and Biotechnology, Hunan Agricultural University, Changsha, China; ^2^ College of Chemistry and Materials, Hunan Agricultural University, Changsha, China; ^3^ Assets and Laboratory Management Department, Hunan Agricultural University, Changsha, China

**Keywords:** brassinosteroids, solid-phase extraction, organ-level distribution, 4-(dimethylamino)-phenylboronic acid, derivatization, online valve-switching

## Abstract

**Background:**

Brassinosteroids (BRs) are a class of naturally occurring steroidal phytohormones mediating a wide range of pivotal developmental and physiological functions throughout the plant’s life cycle. Therefore, it is of great significance to determine the content and the distribution of BRs in plants.Regretfully, although a large number of quantitative methods for BRs by liquid chromatography-tandem mass spectrometry (LC-MS/MS) have been reported, the *in planta* distribution of BRs is still unclear because of their lower contents in plant tissues and the lack of effective ionizable groups in their chemical structures.

**Methods:**

We stablished a novel analytical method of BRs based on C18 cartridge solid-phase extraction (SPE) purification, 4-(dimethylamino)-phenylboronic acid (DMAPBA) derivatization, and online valve-switching system coupled with ultra-high performance liquid chromatography-electro spray ionization-triple quadrupole mass spectrometry (UHPLC-ESI-MS/MS). This method has been used to quantify three structural types of BRs (epibrassinolide, epicastasterone, and 6-deoxo-24-epicastaster one) in different organs of *Brassica napus* L. (rapeseed).

**Results:**

We obtained the contents of three structural types of BRs in various organ tissues of rapeseed. The contents of three BRs in rapeseed flowers were the highest, followed by tender pods. The levels of three BRs all decreased during the maturation of the organs. We outlined the spatial distribution maps of three BRs in rapeseed based on these results, so as to understand the spatial distribution of BRs at the visual level.

**Conclusions:**

Our results provided useful information for the precise *in situ* localization of BRs in plants and the metabolomic research of BRs in future work. The *in planta* spatial distribution of BRs at the visual level has been studied for the first time.

## 1 Introduction

Brassinosteroids (BRs) are a class of growth-promoting steroidal phytohormones widely distributed in the plant kingdom, mediating a wide range of pivotal developmental and physiological functions (Fridman and Savaldi-Goldstein, 2013; Wang et al., 2017; Kim and Russinova, 2020), such as seed germination (Liu et al., 2017), cytodifferentiation (Singh et al., 2021; Takahashi and Umeda, 2022), cell division and expansion (Hacham et al., 2011; Wei and Li, 2016), flowering, pollen germination (Yokota, 1997), reproductive development (Montoya et al., 2005; Bajguz, 2007), modulation of gene expression (Mussig and Altmann, 1999; Neu et al., 2019), maturation, and aging of the plant (Gudesblat and Russinova, 2011). In addition, BRs participate in plants’ tolerance to various abiotic stresses, such as heat (Ahammed et al., 2020), cold (Peres et al., 2019), drought (Marková et al., 2023), salinity (Kolomeichuk et al., 2020; Kong et al., 2021), pesticides (Hou et al., 2018), heavy metals (Samiksha et al., 2016; Sytar et al., 2019), and oxidative stress (Vardhini and Anjum, 2015; Efimova et al., 2018). Moreover, BRs are involved in plant protection against pathogen attacks (Nakashita et al., 2003; Yu et al., 2018). Since BRs were first isolated and identified from rapeseed pollen in the 1970s (Mitchell et al., 1970; Grove et al., 1979), approximately 80 naturally occurring BRs have been identified (Kanwar et al., 2017; Liu et al., 2017; Singh et al., 2021). In the last two decades, exhaustive research has been conducted on these compounds in various processes, such as biosynthesis (Chung and Choe, 2013), metabolism (Neu et al., 2019), signal transduction (Yang et al., 2011), cross-talk with other phytohormones (Saini et al., 2015) and adaptation to environmental stresses (Manghwar et al., 2022; Marková et al., 2023). However, the spatiotemporal distribution of BRs and its effects on coordinated growth and development are still unclear (Fridman and Savaldi-Goldstein, 2013). Although a few reports showed the spatial distribution of BRs in plants (Symons and Reid, 2004; Xin et al., 2013), due to the complex pretreatment steps and ion inhibition in the detection process, the detection sensitivity for BRs was relatively low, and some could not even be detected. The *in planta* spatial distribution map of BRs cannot be comprehensively outlined. Therefore, establishing reliable, highly selective, and sensitive methods for the determination of BRs in plant tissues is highly desirable at this stage.

In order to overcome the difficulties of BR analysis, previous studies focused on developing sample purification methods for BRs from plant matrix, such as liquid–liquid micro-extraction (LLME) (Lv et al., 2014), SPE (Huo et al., 2012; Xin et al., 2013), magnetic solid-phase extraction (MSPE) (Ding et al., 2014; Luo et al., 2017), pipette-tip solid-phase extraction (PT-SPE) (Deng et al., 2016), solid phase micro-extraction (SPME) (Pan et al., 2012), in-line matrix solid-phase dispersion (MSPD)-tandem mixed mode anion exchange (MAX)-mixed mode cation exchange (MCX) SPE (in-line MSPD-MAX-MCX SPE) (Wang et al., 2014), and immunoaffinity chromatography (IAC) (Oklestkova et al., 2017). However, most of these methods were complicated, requiring lengthy sample pretreatment; in particular, some materials need to be synthesized or assembled by the authors themselves, which increases the technical difficulty and uncertainty of the experiment, resulting in poor repeatability and lower recovery rate.

To simplify the experimental procedures, some online pretreatment methods have been developed, such as online polymer monolith microextraction and *in situ* derivatization (PMME-ISD) (Wang et al., 2020) and online two-dimensional microscale solid phase extraction-on column derivatization (2DμSPE-OCD) (Wu et al., 2013). These methods require that the SPE materials are firstly filled into a monolithic column, as well as connected with pumps, high-pressure rotary valve, and analytical instrument *via* poly ether-ether-ketone (PEEK) transfer line. The pretreatment and analysis of the samples were completed by changing the flow path of the mobile phase. Although these automated pretreatment methods can reduce the influence of artificial factors on the determination results, some tough operations such as purchasing specific LC pumps and samplers or building self-made SPE purification columns and automated operation platform would be difficult for the general experimental operators. In addition, the consistency of such instruments is usually not comparable to commercial ones, and the reproducibility of different batches of samples has not been investigated in these studies.

In this study, we firstly chose C_18_ cartridges that available in the market as SPE purification cartridges. Based on the hydrophobicity of BRs and the separation principle of LC, we could remove a large amount of higher polar interfering substances by cleaning the cartridges with 50% methanol before eluting BRs with 100% methanol, and obtain higher-purity BR extracts. BRs lacked ionizable groups, which caused lower sensitivity of BRs analyzed by LC-MS (Chu et al., 2017). Fortunately, BRs are vicinal diol-containing compounds and have several hydroxyl groups. The specific boronic acid-diol reaction can be fully appreciated to more sensitively develop derivatization methods for BRs. Several boronic acid reagents used for the derivatization of BRs have been reported, such as 2-bromopyridine-5-boronic acid (BPBA) (Huo et al., 2012), m-aminophenylboronic acid (m-APBA) (Wu et al., 2013; Wang et al., 2014), 4-mercaptophenylboronic acid (4-MPBA) (Chen et al., 2018), 3(4)-(dimethylamino)-phenylboronic acid (DMAPBA) (Xin et al., 2013; Ding et al., 2014; Chu et al., 2017; Luo et al., 2017), 4-phenylaminomethyl-benzeneboric acid (4-PAMBA) (Yu et al., 2016), 4-borono-N,N,N-trimethylbenzenaminium iodide (BTBA) (Deng et al., 2016), 2-(4-boronobenzyl) isoquinolin-2-ium (BBII) (Luo et al., 2018), 2-methyl-4-phenylaminomethylphenylboronic acid (2-methyl-4-PAMBA) (Wang et al., 2020), and rhodamine B-boronic acid (RhB-BA) (An et al., 2020). Here, we chose DMAPBA as the derivatization reagent because of its commercial availability, high MS response intensity (Xin et al., 2013), and more usage reports in BR research (Ding et al., 2014; Chu et al., 2017; Luo et al., 2017). In addition, we selected a high-pressure six-port rotary valve as an online valve-switching system installed in front of the electrospray ionization (ESI) source of the mass spectrometer for minimizing the amount of interfering substances entering the mass spectrometer. In this way, not only was the influence of interfering substances and sample matrix on the ionization efficiency of BRs decreased, but the pollution of ESI ion source and ion channel was reduced as well, and the sensitivity and the repeatability of BRs were improved.

The selection of model molecules is also a problem worth exploring because of the wide variety of BRs. BRs are usually grouped into C_27_, C_28_, and C_29_ forms based on carbon numbers in their structures. Among the three subgroups, C_28_-BRs are ubiquitous in the plant kingdom and represent the most active form of naturally occurring BRs (Bajguz and Tretyn, 2003), which represent approximately 60% from the total identified BRs and intermediates (Kanwar et al., 2017). Epibrassinolide (epiBL), epicastasterone (epiCS), and 6-deoxo-24-epicastasterone (d-epiCS) are commonly used as standards, representing three structural types of C28-BRs. In addition, although BRs are usually grouped into C_27_, C_28_, and C_29_ types, the structures formed by C1–C27 in the three subgroups are the same. Thus, we tested only epiBL, epiCS, and d-epiCS in this study.

Collectively, we established a new BR quantitative method based on the combination of C_18_ cartridge SPE purification, DMAPBA derivatization, and online valve-switching system coupled with UHPLC-ESI-MS/MS. Then, we have accurately quantified the contents of three structural types of BRs in various organs of rapeseed by this method, thereby revealing the organ-level distribution of BRs in rapeseed. The results provide a useful scheme for the *in planta* spatial distribution of BRs and have reference value for studying the signaling pathway and regulatory mechanism of BRs in plants.

## 2 Materials and methods

### 2.1 Chemicals and reagents

EpiBL (C_28_H_48_O_6_, purity > 98%), epiCS (C_28_H_48_O_5_, purity > 98%), d-epiCS (C_28_H_50_O_4_, purity > 98%), [^2^H_3_]brassinolide ([^2^H_3_]BL, C_28_H_45_^2^H_3_O_6_, purity > 95%), and [^2^H_3_]castasterone ([^2^H_3_]CS, C_28_H_45_^2^H_3_O_5_, purity > 94%) were purchased from Olchemim Ltd. (Olomouc, Czech Republic). All of the BRs and their stable isotope analogue stock solutions were prepared at 500 μg mL^−1^ in acetonitrile at −20°C.

Chromatography grade acetonitrile (CH_3_CN) and methanol (CH_3_OH) were purchased from TEDIA Co. (Ohio, USA) and Merck (Darmstadt, Germany), respectively. 4-(Dimethylamino)-phenylboronic acid (DMAPBA, C_8_H_12_BNO_2_, purity>95%) and formic acid (CH_2_O_2_) were obtained from TCI Development Co., Ltd. and Sinopharm Chemical Reagent Co., Ltd. (Shanghai, China), respectively. C_18_ cartridges (Sep-Pak ^®^ Vac 1cc, 100 mg) were purchased from Waters Corporation (Delaware, USA). Ultrapure water (resistivity ≥ 18.2 MΩ/cm) used throughout the study was purified by the Simplicity-UV water purification system (Merk Millipore, USA).

### 2.2 Plant materials

**Figure 1:**
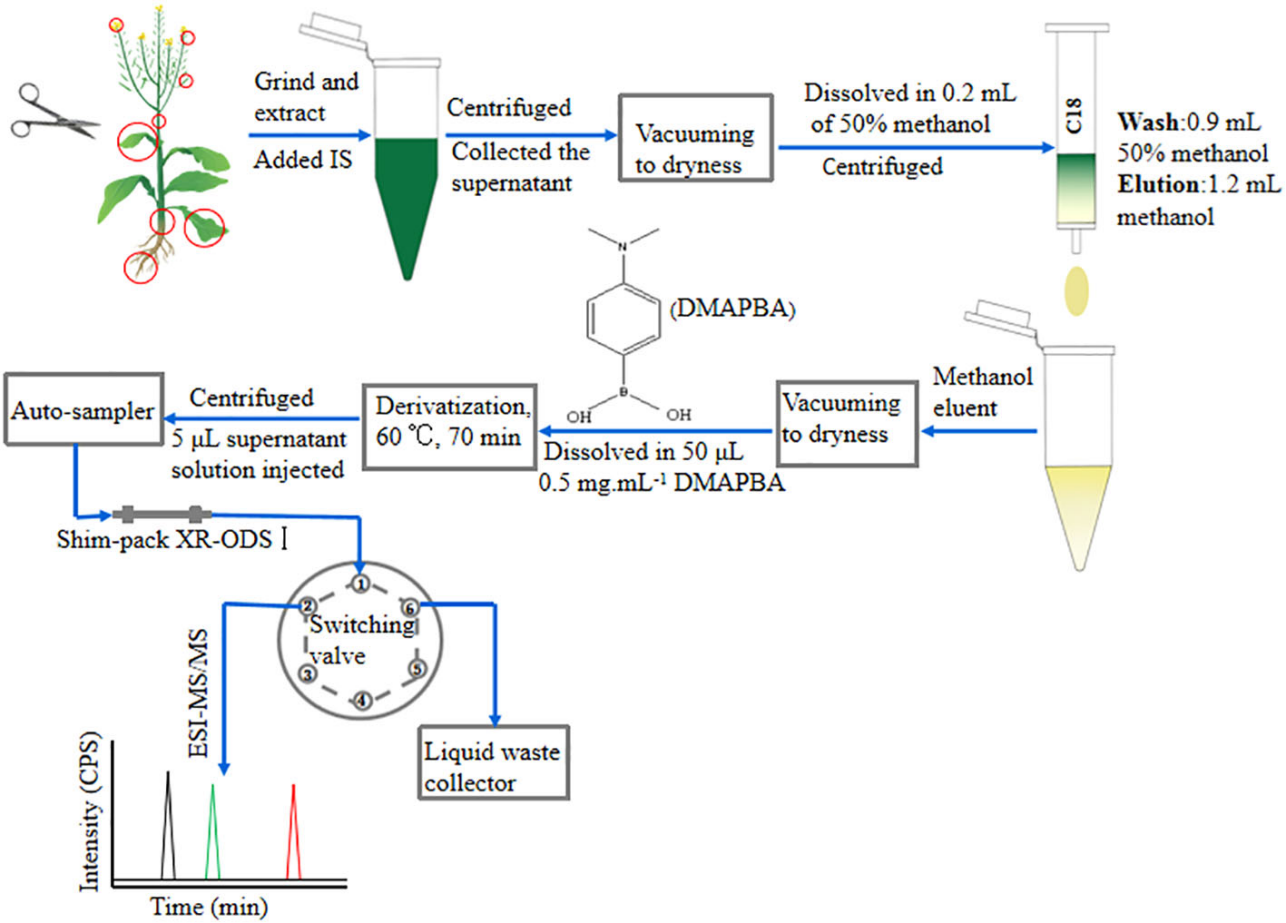
Schematic diagram of the high-sensitivity scheme operation for quantitative analysis of BRs. The red circles indicated the sampling site. IS, internal standards; CPS, counts per second.

Seeds from *Brassica napus* line L104 (No. L104) were grown in paddy soil in the experimental base of Hunan Agricultural University (Changsha, China) during the 2021–2022 growing seasons. Seedlings were randomly transplanted into plastic pots with a diameter of 30 cm and a height of 40 cm in the net house, one pot for each plant, at a row spacing of 50 cm. Compound fertilizer (N:P:K = 1:1:1) was applied at a rate of 8 g per plant as base fertilizer and urea was applied at 3 g per plant as dressing fertilizer, with watering to keep the soil moist. The field management followed the standard agricultural practice. L104 rapeseed seedlings were sampled during the fruiting period to obtain organ-level distribution of BRs; 18 rapeseed seedlings with good growth were selected as experimental materials and were divided into three groups (six plants per group) as three biological replicates. As shown in **Figure 1**, roots, tender stems, old stems, young leaves, old leaves, tender pods, mature pods, and fully expanded flowers were isolated by sharp surgical scissors, collected and wrapped in tin foil, respectively, quickly frozen in liquid nitrogen, and stored in a −80°C ultralow-temperature refrigerator (DW-86L388J, Shandong, China) to reduce the effect of stress response such as mechanical stress on BR levels.

### 2.3 Derivatization of BRs with DMAPBA

EpiBL, epiCS, d-epiCS, [^2^H_3_]BL, and [^2^H_3_]CS were used to perform the derivatization experiment. Five micrograms of each BR was dissolved in 1 mL of 0.5 mg mL^−1^ DMAPBA acetonitrile solution. The reaction solutions were vortexed (SI HYQ3110, USA), and kept in a thermostatic water bath (HC21006, Chongqin, China) at 60°C for 70 min. The derivatives were centrifuged at 15,000*g* for 10 min (Eppendorf centrifuge 5418, Germany), and stored at −20°C for further analysis.

### 2.4 Preparation of plant samples

The extraction and purification of BRs had been improved (Bajguz et al., 2019; Liu et al., 2022). Briefly, fresh plant tissues were ground to a fine powder under liquid nitrogen. Then, 100-mg of plant tissue powder was accurately weighed into a 2-mL centrifuge tube. [^2^H_3_]BL (0.5 ng) and [^2^H_3_]CS (0.5 ng) were added into the sample serving as internal standards. Methanol (1 mL) was added to extract BRs at 4°C overnight. Each sample consisted of three technical replicates. Supernatant was collected after centrifugation at 15,000*g*, 4°C for 10 min (Eppendorf centrifuge 5415R, Germany). The residue was mixed with 0.5 mL of methanol, and was centrifuged after 2 h. The supernatant was also collected and added into the previous supernatant. The collected supernatant was vacuumed to dryness in a Jouan RCT-60 concentrator (Jouan, France), and then reconstituted in 200 μL of 50% methanol for C_18_ cartridge SPE purification. The C_18_ cartridge SPE purification procedures were selected with the following three steps in sequence: (1) the supernatant was loaded into a C_18_ SPE cartridge after centrifugation, (2) 900 μL of 50% methanol was used as eluent for washing the C_18_ SPE cartridge, and (3) 1.2 mL of methanol was used as eluent eluting and collecting BRs adsorbed on the cartridge. The eluent was vacuumed to dryness again, dissolved in 50 μL of 0.5 mg mL^−1^ DMAPBA acetonitrile solution to perform the derivatization.

### 2.5 Online valve-switching system

An FCV-20AH_2_ high-pressure six-port rotary valve (Shimadzu, Japan) was used as an online valve-switching system. It was installed between a Shim-pack XR-ODSI(2.0 mm I.D. × 75 mm, 2.2 μm) column and an electrospray ionization (ESI) source, *via* a 500 mm × 0.1mm I.D. poly (ether-ether-ketone) (PEEK) connection for switching the mobile phase between the mass spectrometer and the waste liquid collector. The valve can be set to automatic switching flow path of the mobile phase. In this study, the valve was switched to the 1–2 position at 9–12.6 min and 17–18.2 min, and the mobile phase took BRs-DMAPBA entering into the mass spectrometer to be detected. At other times, the valve was switched to the 1–6 position, and the mobile phase took the sample matrix and interfering substances entering the waste liquid collector. The valves’ 3–5 position were blocked with dead plugs.

### 2.6 UHPLC-ESI-MS/MS conditions

Analysis of BRs was performed on a UHPLC-ESI-MS/MS system consisting of a Shimadzu MS-8030 Plus mass spectrometer (Japan) with an ESI source, a Shimadzu LC-20AD UHPLC system (Japan) with two 20AD XR pumps, a SIL-20A XR auto-sampler, a CTO-20AC thermostat column compartment, and a DGU-20A3R degasser. Data acquisition and processing were performed with LabSolution 5.42 SP4 software.

The UHPLC separation was performed at 35°C on a C_18_ column (Shimadzu, Shim-pack XR-ODSI 2.0 mm I.D. × 75 mm, 2.2 μm). A 22-min gradient of 0.1% formic acid in H_2_O (A) and methanol (B) was employed for the separation of BRs-DMAPBA with a flow rate of 0.25 mL min^−1^. A linear gradient with the following proportions (v/v) of solvent B was applied: 0–15 min at 50%–100%, 15–19 min at 100%, followed by 3 min of re-equilibration at 50%. The injection volume was 5 μL.

BRs-DMAPBA was quantified by multiple reaction monitoring (MRM) in the positive mode. BRs-DMAPBA (epiBL, epiCS, d-epiCS, [^2^H_3_]BL, and [^2^H_3_]CS) at 5 μg mL^−1^ was employed to scan and optimize the MRM parameters. EpiBL-DMAPBA at 100 ng mL^−1^ was employed to optimize the ESI source parameters. The optimal MRM parameters for BRs-DMAPBA are listed in **Table 1**. The optimal conditions for ESI source parameters were as follows: desolvation (DL) temperature, 200°C; heat block temperature, 400°C; nebulizing gas, 2.5 L min^−1^; drying gas, 12 L min^−1^; capillary voltage, 4.5 kV.

**Table 1:** Optimized MRM parameters for BRs-DMAPBA (Q_1_ and Q_3_ pre bias [V]; CE [eV]).

Analyte	Quantification				Confirmation			
	Q_1_/Q_3_ (m/z)	Q_1_ pre bias	CE	Q_3_ pre bias	Q_1_/Q_3_ (m/z)	Q_1_ pre bias	CE	Q_3_ pre bias
epiBL-DMAPBA	610.3/190.3	−30	−43	−20	610.3/176.2	−30	−49	−24
epiCS-DMAPBA	594.1/176.2	−30	−55	−17	594.1/190.25	−30	−49	−25
d-epiCS-DMAPBA	580.1/176.25	−28	−55	−17	580.1/190.3	−28	−47	−19
[^2^H_3_]BL-DMAPBA	613.4/190.2	−32	−44	−19	613.4/176.2	−32	−55	−17
[^2^H_3_]CS-DMAPBA	597.1/176.2	−30	−55	−17	597.1/190.25	−30	−49	−25

### 2.7 Method validation

The linearity of the proposed method was evaluated by different concentrations of epiBL, epiCS, and d-epiCS standards (1, 2.5, 5, 10, 25, 50, 100, 200, and 400 ng mL^−1^) with a fixed concentration of internal standards (IS, [^2^H_3_]BL, and [^2^H_3_]CS 10 ng mL^−1^, respectively). The calibration curves of epiBL and epiCS were constructed by plotting the peak area ratios (analyte/IS) versus the concentration of epiBL and epiCS (IS concentration was considered as 1), respectively. The calibration curve of d-epiCS was constructed by plotting the peak area versus the concentration of d-epiCS.

The precision and accuracy of the proposed method were evaluated by spiking epiBL, epiCS, and d-epiCS standards (5, 20, and 200 ng mL^−1^) into 100-mg tender pod sample extracts in triplicate and then treated with the proposed procedure described above. Relative recoveries of the whole method were calculated according to the linear curves generated from the standards in matrix-free solvent.

The matrix effects (MEs) of the proposed method were evaluated by spiking IS [^2^H_3_]BL (0.5 ng) and [^2^H_3_]CS (0.5 ng) into 100-mg tender pod sample extracts. The spiked samples were divided into three groups, with each group consisting of three replicates. The first group was only derivated, the second group was purified by C_18_ cartridge SPE and derivated, and the third group was purified by C_18_ cartridge SPE and derivated, and the mobile phase was switched into the waste liquid collector during the non-detection period. The peak areas of [^2^H_3_]BL and [^2^H_3_]CS in these samples detected by UHPLC-ESI-MS/MS were compared with those of [^2^H_3_]BL and [^2^H_3_]CS in acetonitrile, and the MEs of [^2^H_3_]BL and [^2^H_3_]CS were calculated.

The proposed method was also used for analysis of BRs in various rapeseed organs. To ensure the accuracy of the experimental results, three biological replicates and three technical replicates were set up for each organ tissue, respectively. Briefly, fresh roots, tender and old stems, young and old leaves, flowers, and tender and mature pods of rapeseed were ground into powder in liquid nitrogen, and 100-mg of rapeseed sample powder was accurately weighed into a 2-mL centrifuge tube and extracted by methanol, then treated with the proposed procedure as described above.

## 3 Results

### 3.1 Optimization of derivatization conditions

**Figure 2:**
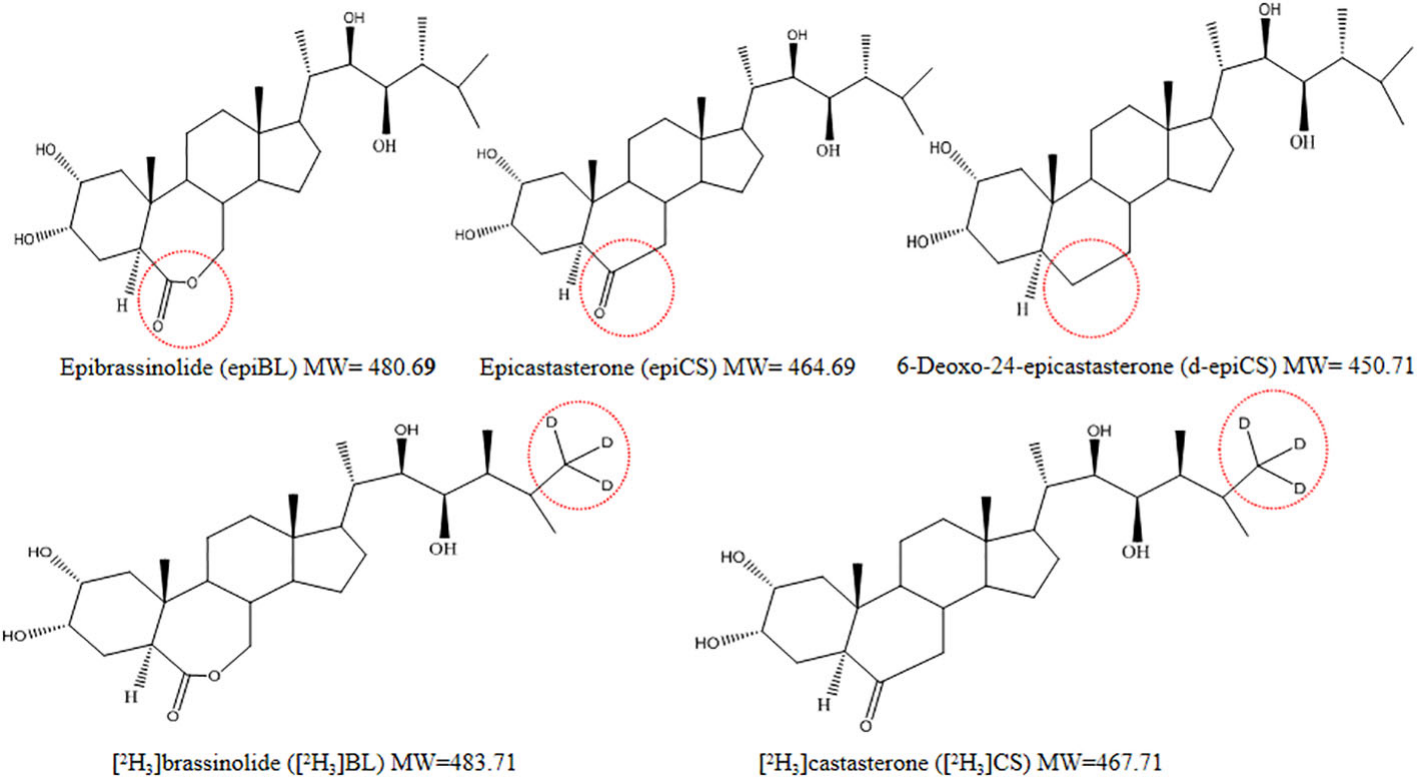
Chemical structures of three BRs and two stable isotope analogues of BRs. The red circles indicate the three different substituents (7-oxalactone, 6-oxoketone, and 6-deoxo types) in the B-ring and the deuterium-labeled locations of the two isotopic analogues.

**Figure 3:**
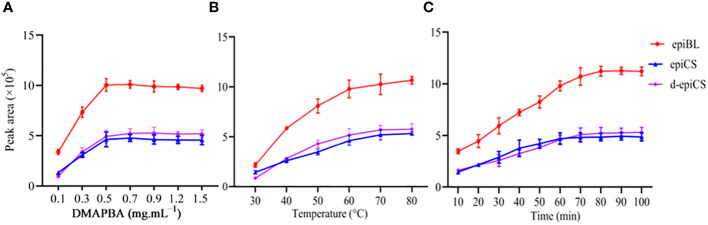
Optimization of derivatization reaction conditions for three BRs by DMAPBA. **(A)** The concentration of DMAPBA. **(B)** Reaction temperature. **(C)** Reaction time. These data are the means ± SDs (*n* =3).

EpiBL, epiCS, and d-epiCS were optimized for derivative experiments. Their structures are almost identical except for the parts marked with red circles, with only one-O or two-O difference (**Figure 2**). To improve the MS response of BRs, we chose DMAPBA as the derivatization reagent. In order to optimize the derivational efficiency of BRs, we compared the peak areas of three BRs-DMAPBA (epiBL-DMAPBA, epiCS-DMAPBA, and d-epiCS-DMAPBA) standards by MRM in the positive mode under different derivatization conditions, including the concentration of DMAPBA, reaction time, and reaction temperature (**Figure 3**). Considering the unstable lactone structure of BRs at high temperature and the boiling point of anhydrous acetonitrile (81–82°C), 0.5 mg mL^−1^ DMAPBA dissolved in anhydrous acetonitrile, a reaction temperature of 60°C, and a reaction time of 70 min were selected as the later derivatization conditions.

### 3.2 Optimization of C_18_ cartridge SPE purification procedures

BRs are a category of natural polyhydroxysteroidal lactones/ketones compounds with high hydrophobicity and neutral property (Bajguz, 2011). We selected Waters Sep-Pak ^®^ Vac 1cc (100-mg) C_18_ SPE cartridges as pretreatment purification cartridges because of their unique physicochemical properties. Firstly, 5 μL of mixed standard solution of three BRs at a concentration of 100 ng mL^−1^was injected into UHPLC-ESI-MS/MS and detected in selective ion monitoring (SIM) mode to investigate their retention time and elution conditions separated by a C_18_ LC column. The selected ion chromatograms (SIC) are shown in **Supplementary Figure 1**. EpiBL had the shortest retention time, while d-epiCS had the longest retention time. Therefore, epiBL and d-epiCS were selected as model compounds to optimize the C_18_ cartridge SPE purification procedure. Subsequently, 200 μL of mixed standard solution of epiBL and d-epiCS (50% methanol) at a concentration of 100 ng mL^−1^ was loaded into a C_18_ cartridge. In order to obtain higher-purity BRs extract, we cleaned the C_18_ cartridges with 50% methanol to remove polar interfering substances. At the same time, the signal intensity of epiBL and d-epiCS standards per 100 μL of eluent was detected by UHPLC-ESI-MS/MS. When the ionic signal of epiBL could be detected, we used 100% methanol to elute the BRs adsorbed on the cartridge. By the same method, the signal intensity of epiBL and d-epiCS per 100 μL of methanol eluent was investigated until no ionic signal of d-epiCS was detected to obtain the maximum recovery rate. Finally, the following C_18_ cartridge SPE purification procedure optimized conditions were selected: (1) the sample solution was 50% methanol with a volume of 200 μL, (2) the cleaning solution was 50% methanol with a volume of 900 μL, and (3) the eluent was 100% methanol with a volume of 1.2 mL. As can be seen from the operation procedure (**Figure 1**), it is a simple, fast, low-cost, and environmentally friendly protocol for BR purification.

### 3.3 Optimization of chromatographic separation conditions

Firstly, a Shim-pack XR-ODSI (2.0 mm I.D. × 75 mm, 2.2 μm) was chosen to perform the separation of three BRs-DMAPBA by comparing the resolution, the retention time, and the peak shape of BRs-DMAPBA on different columns. Then, we compared the signal intensity of epiBL-DMAPBA with methanol/water and acetonitrile/water used as the mobile phase, and found the signal intensity in the methanol/water system to be approximately five times than that of the acetonitrile/water system (**Supplementary Figure 2**). Therefore, methanol/water was selected as the mobile phase for subsequent experiments. For the investigation of additives in mobile phase, the best signal intensity and signal-to-noise ratio (S/N) were obtained when 0.1% formic acid was added by comparing the signal intensity and S/N of the effects on 0.02%, 0.05%, and 0.1% formic acid as well as acetic acid aqueous solution. Subsequently, we compared the signal intensity of epiBL-DMAPBA with isocratic elution of different methanol proportions, and found that the signal intensity of epiBL-DMAPBA was enhanced with the increase of methanol proportion in the mobile phase. In order to minimize the influence of co-elution interfering substances on the quantification of BRs-DMAPBA, gradient elution was chosen. Finally, a linear gradient with the following proportions (v/v) of solvent methanol was selected: 0–15 min at 50%–100% and 15–19 min at 100% by comparing further experiments.

### 3.4 Installation of an online valve-switching system

DMAPBA can not only react with BRs in plant extracts to produce BRS-DMAPBA, but also react with cis-diol-containing interferents to produce interfering substances similar to BRs-DMAPBA (Ding et al., 2014). If residual DMAPBA, BRs-DMAPBA analogues, and other interfering substances enter the ESI-MS/MS, the ESI source and ion channels would be seriously polluted, and the ionization efficiency and signal intensity of BRs-DMAPBA would be affected. Therefore, we installed a high-pressure six-port rotary valve in front of the ESI source. A large amount of interfering substances could be switched into the waste liquid collector (**Figure 1**). The working procedure was as follows: (1) 5 μL of the BRs-DMAPBA standard solution was injected into UHPLC-ESI-MS/MS, and the mobile phase took the BRs-DMAPBA through valves’ 1–2 position to the mass spectrometer for detecting the retention time of each BRs-DMAPBA; (2) the times of valve-switching were set in the analytical method according to the retention time of each BRs-DMAPBA. In this study, in order to minimize the amount of sample matrix entering the mass spectrometer but not affecting the data acquisition, the times of valve-switching were chosen as follows: (1) switching to the 1–2 position at 9–12.6 min and 17–18.2 min for detecting BRs-DMAPBA, (2) switching to the 1–6 position at other times for removing interfering substances. This method not only simplified the sample preparation process, but also reduced contamination of ESI source and ion channels; thus, the sensitivity and repeatability of BRs-DMAPBA were improved.

### 3.5 Optimization of mass spectrometer conditions

Generally, MRM parameters need to be optimized prior to LC-MS analysis for MRM-MS analysis of analytes, including precursor ion, product ion, and collision-induced dissociation (CID) voltage. In this study, we used the concentration of 5 μg mL^−1^ of BRs-DMAPBA single standard solution for full scan and obtained their precursor ions. We obtained the parameters of automatically optimized MRM such as quadrupole1 (Q_1_), quadrupole3 (Q_3_), and collision energy (CE) voltage using the “Optimization for Method” data acquisition software. The MRM parameters of the established UHPLC-ESI-MS/MS method are shown in **Table 1**. Next, 5 μL of epiBL-DMAPBA with a concentration of 100 ng mL^−1^ was injected into UHPLC-ESI-MS/MS. The signal intensity of epiBL-DMAPBA was investigated in MRM mode with different ion source parameters including capillary voltage, DL temperature, heat block temperature, nebulizing gas flow, and drying gas flow. Thus, the best ion source parameters were obtained.

**Figure 4:**
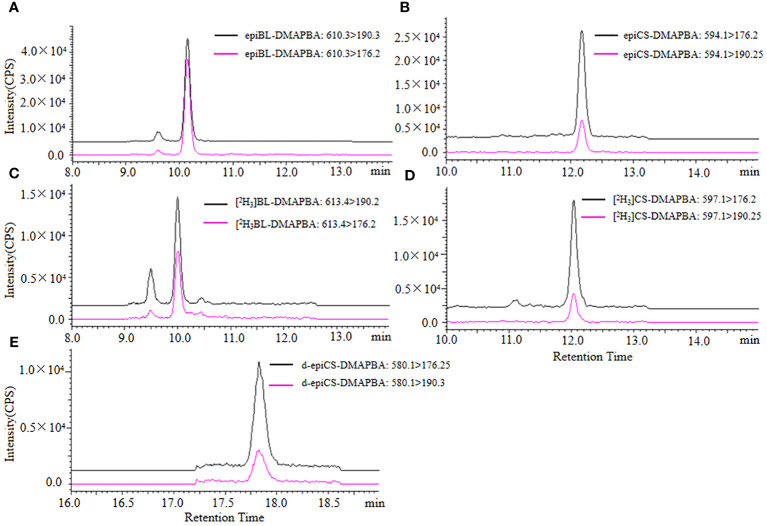
The extraction ion chromatograms of epiBL-DMAPBA **(A)**, epiCS-DMAPBA **(B)**, [^2^H_3_]BL-DMAPBA **(C)**, [^2^H_3_]CS-DMAPBA **(D)**, d-epiCS-DMAPBA **(E)** by UPLC-ESI-MS/MS.

**Figure 5:**
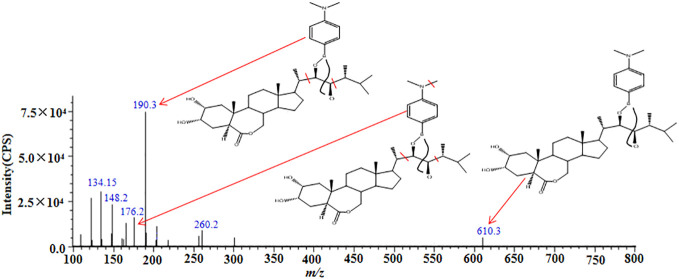
MS/MS spectra and proposed fragment pathways of epiBL-DMAPBA.

We obtained the extraction ion chromatograms (EIC) of three BRs-DMAPBA and their two internal standards by the optimized MRM method (**Figure 4**). We investigated the MS fragmentation pathway of BRs-DMAPBA by product ion scan. Despite the fact that these BRs added an easy ionizing group by derivatization, only their precursor ions produced by ESI ionization were different, and their characteristic ions produced by collision-induced ionization are the same, including the two isotope internal standards. The fragmentation pattern is shown in **Figure 5**.

### 3.6 Evaluation of the matrix effect

**Figure 6:**
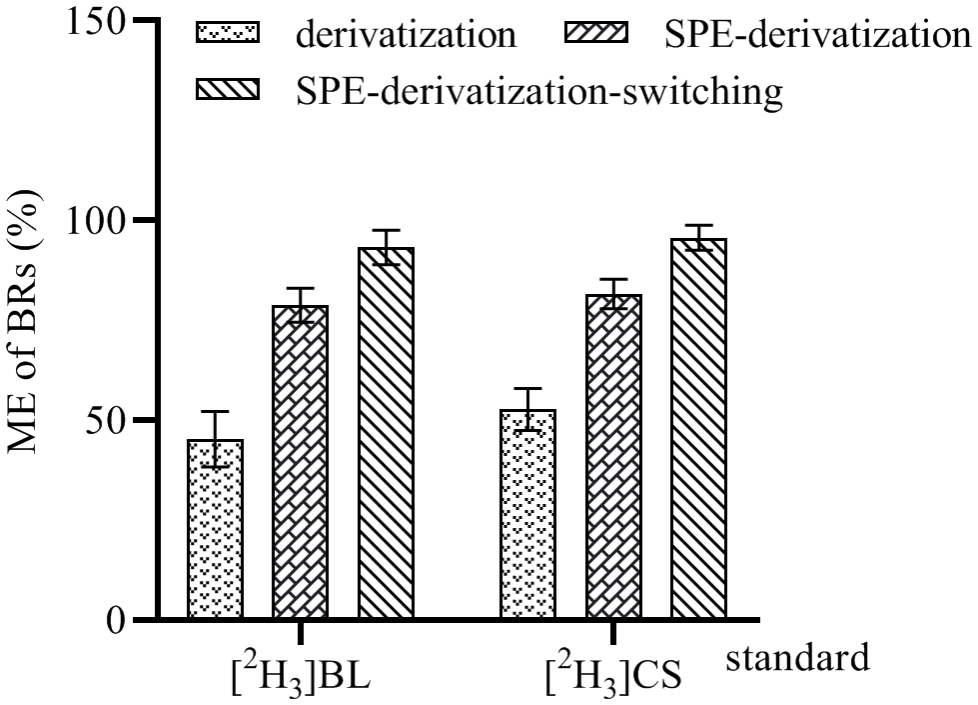
Matrix effect (ME) of 100-mg tender pod samples of rapeseed analyzed by UHPLC-ESI-MS/MS. These data are the means ± SDs (*n* = 3).

A complex sample matrix may reduce or enhance the ionization of the analytes in ESI, thereby affecting the MS signal (Constantopoulos et al., 1999; Yarita et al., 2015). In order to evaluate the MEs of this method, 0.5 ng of [^2^H_3_]BL and [^2^H_3_]CS standards was spiked into rapeseed tender pod samples (100-mg) and methanol to be used for the test. The spiked samples were divided into three groups, with each group consisting of three replicates. The first group was only derivated, the second group was purified by C_18_ SPE cartridge and derivated, and the third group was purified by C_18_ SPE cartridge and derivated, and the mobile phase during the non-detection period was switched into the waste liquid collector. The peak areas of [^2^H_3_]BL-DMAPBA and [^2^H_3_]CS-DMAPBA in all samples analyzed by UHPLC-ESI-MS/MS were compared with those in acetonitrile. MEs were calculated as follows: the peak areas of [^2^H_3_]BL-DMAPBA and [^2^H_3_]CS-DMAPBA in the rapeseed tender pod extracts were divided by their peak areas in acetonitrile, respectively. As shown in **Figure 6**, in the first group, although the response of BRs was improved after derivatization, the interfering substances had a strong ion inhibition detected for the BRs-DMAPBA. In the second group, although the samples were purified by C_18_ cartridge SPE, and a large amount of sample matrix was removed, there were still interfering substances such as the residue of derivative reagents and BRs-DMAPBA analogues, which had an ionic inhibition effect on the detection of BRs-DMAPBA. In the third group, the mobile phase only near the retention time took BRs-DMAPBA into the mass spectrometer through the online valve-switching system. Most of the matrix and interfering substances were removed, and the MEs reached 93.25% ([^2^H_3_]BL) and 95.67% ([^2^H_3_]CS), indicating that the C_18_ cartridge SPE purification coupled with the use of online valve-switching system minimized ion inhibition.

### 3.7 Method evaluation

For quantification of three BRs in the sample by analysis of 5-μL BRs-DMAPBA standard solutions, nine level calibration plots (1–400 ng mL^−1^) were carried out for the whole method. The BRs-DMAPBA standard solutions contained internal standard (IS) [^2^H_3_]BL and [^2^H_3_]CS with the concentration of 10 ng mL^−1^. As shown in **Figure 4**, epiBL-DMAPBA and epiCS-DMAPBA almost had the same retention times as internal standards [^2^H_3_]BL-DMAPBA and [^2^H_3_]CS-DMAPBA, respectively. The calibration curves of epiBL-DMAPBA and epiCS-DMAPBA were constructed by plotting the peak area ratios (analyte/IS) versus BR (epiBL and epiCS) concentrations. However, d-epiCS-DMAPBA was quantified by the external standard method because of no suitable internal standard. The calibration curve of d-epiCS-DMAPBA was constructed by plotting the peak areas versus d-epiCS concentrations. As shown in **Table 2**, good linearities were obtained, and the correlation coefficients (*R* ^2^) were all better than 0.9986. The LOD and LOQ values were calculated at a signal-to-noise ratio (S/N) of 3 and 10 times, respectively. LODs and LOQs were in the range of 0.3–2.5 ng mL^−1^and 1.0–8.3 ng mL^−1^, respectively. The results showed that it was quite sensitive for profiling BRs in plant samples.

**Table 2:** Linear regression equation and LOD data of BRs analyzed by UHPLC-ESI-MS/MS.

Analyte	Linear range (ng mL^−1^)	Equation of linear regression	*R* ^2^	LOD^a^ (ng mL^−1^)	LOQ^b^ (ng mL^−1^)
epiBL	1–400	*Y* = 0.0601*x*+0.2224	0.9986	0.3	1.0
epiCS	1–400	*Y* = 0.0137*x−*0.0492	0.9997	0.5	1.7
d-epiCS	1–400	*Y* = 684.1091*x−*933.7025	0.9974	2.5	8.3

a LOD, limit of detection.

b LOQ, limit of quantification.

In order to evaluate the accuracy and precision of this method, extracts of rapeseed tender pod samples (100-mg) spiked with standards at three different concentrations (5, 20, and 200 ng mL^−1^ BRs/10 ng mL^−1^ [^2^H_3_]BL and [^2^H_3_]CS) were employed for the test. The intra-day precisions were evaluated by repeating the process three times within 1 day, and the inter-day precisions were investigated on three successive days. The relative recoveries were in the range of 90.42%–101.82%, and the relative standard deviations (RSDs) of intra- and inter-day precision were below 10.33% (**Table 3**). The results indicated a good reproducibility and accuracy of the method.

**Table 3:** Accuracy and precision (intra- and inter-day) for the determination of BRs in rapeseed samples (100-mg fresh mass).

Analyte	Intra-day precision (%, *n* = 3)^a^			Inter-day precision (%, *n* = 3)^a^			Recovery (%, *n* = 3)^a^		
	Low	Medium	High	Low	Medium	High	Low	Medium	High
epiBL	4.95	3.51	6.42	8.24	6.92	7.43	94.51	93.87	95.26
epiCS	8.21	4.25	4.87	3.88	4.65	8.67	92.57	96.45	93.53
d-epiCS	9.54	7.53	8.62	7.66	8.69	10.33	101.82	90.42	92.79

a BR standards were spiked in rapeseed samples at three different concentrations (5, 20, and 200 ng mL^−1^)

### 3.8 The organ-level distribution measurement of BRs in rapeseed

**Figure 7:**
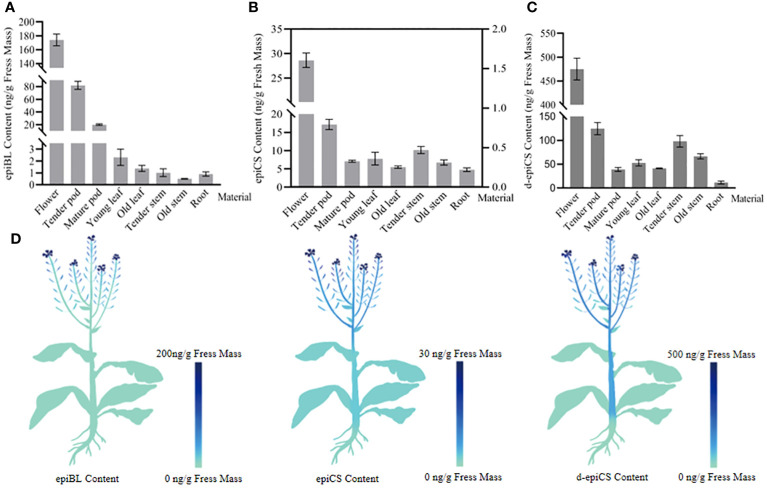
The contents of epiBL **(A)**, epiCS [**(B)**, the contents of epiCS in flowers and fruit pods were represented by the left ordinate; others were represented by the right ordinate], and d-epiCS **(C)** measured in various rapeseed organs, and the diagrammatic spatial distribution of three BRs is outlined **(D)**. The data are the means ± SDs (*n* = 9).

The quantification of BRs in different organs of rapeseed was performed using the established method. In order to obtain the organ-level distribution of BRs in rapeseed, we divided the rapeseed organ categories in detail and analyzed the contents in 100-mg of various organ tissues. As shown in **Supplementary Figure 3**, three BRs-DMAPBA could be detected in fresh rapeseed samples, and their retention times were almost the same as the standard and internal standard, validating the high selectivity and the high sensitivity of our method. **Figures 7A–C** show the content of three BRs in different organ tissues of the rapeseed. The figures show that the contents of three BRs are all the highest in flowers, followed by tender pods. Among them, the contents of epiBL and epiCS gradually decreased from top to bottom along the trunk. Interestingly, the levels of three BRs all decreased as the organ tissues mature. In addition, the diagrammatic map outlined the spatial distribution of epiBL, epiCS, and d-epiCS in rapeseed according to the results detected by UHPLC-ESI-MS/MS (**Figure 7D**). It provided a spatial framework of BRs in various parts of rapeseed, and contributed to the understanding for precise *in situ* localizations of BRs in plants.

### 3.9 Method comparison

In this study, we established a simple and easy way to measure the levels of BRs. Compared to the reported methods, we have made three adjustments in this study. First, the mobile phase without any other chemical reagent was used to elute the C_18_ cartridge SPE purification processes, which significantly decreased the ion inhibition effect of interfering substances on the target substance with low abundance. Second, DMAPBA, a highly efficient and inexpensive derivative reagent, was used to improve the sensitivity of BR detection. Third, precise control of mobile phase entering the mass spectrometer was obtained by installing an online valve-switching system, which helped to separate target substances from interfering substances and avoid the pollution of the mass spectrometer. In addition, we also compared our proposed method in terms of the pretreatment method, technical difficulties, the content of BRs detected, the amount of plant tissues, and internal standard with representative methods published in the last few years (**Supplementary Table 1**) (Huo et al., 2012; Wu et al., 2013; Xin et al., 2013; Ding et al., 2014; Wang et al., 2014; Deng et al., 2016; Luo et al., 2018; An et al., 2020; Wang et al., 2020). It can be seen that we encountered fewer technical difficulties in the proposed method. Owing to the simplification of the sample pretreatment process and the use of the online valve-switching system, the sensitivity and repeatability have been significantly improved, and the contents of three BRs in various organs of the rapeseed have been detected successfully. The contents of three BRs in flowers of rapeseed are higher (Luo et al., 2018; An et al., 2020), but lower in leaves (Huo et al., 2012; Wu et al., 2013; Ding et al., 2014; Deng et al., 2016), which is consistent with the previous reports. In this study, in order to obtain the organ-level distribution of BRs in rapeseed, a relatively large amount of samples (100 mg) was selected. Thereby, the proposed method can be applied to the determination of BRs in almost all plant tissues for the wide universality.

## 4 Discussion

BRs are a class of steroid phytohormones that can regulate the plant growth and development at the micro-level concentration (María et al., 2017). Recent studies have found that BRs can induce callus formation and differentiation (Singh et al., 2021), regulate plant architecture (Xia et al., 2021), improve crop yield and quality (Vriet et al., 2012; Anwar et al., 2018; Chmur and Bajguz, 2021), enhance plant tolerance to environmental stresses (Bajguz and Hayat, 2009; Kanwar et al., 2012; Hafeez et al., 2021; Kong et al., 2021), and decrease pesticide residues (Hou et al., 2018). Moreover, BRs can reduce the negative effect of damaging environmental factors on plants, improve their adaptability to adverse environmental conditions (Zhu et al., 2016; Kolomeichuk et al., 2020), and have proven their protective effect on plants growing under various stresses (Khripach et al., 2000; Krishna, 2003; Ahammed et al., 2020; Singh et al., 2016; Vardhini and Anjum, 2015). At present, BRs have been regarded as effective and ecofriendly natural stress-resistant growth regulators, and have great application prospects in future agricultural production (Liu et al., 2017; Ahammed et al., 2022). Therefore, it is of great significance to study the accurate quantitative determination method for BRs. In recent years, with the development of LC-MS/MS, the selectivity and sensitivity of analytical methods have been improved, and the accurate quantification of trace organic compounds such as BRs has become possible. However, the absence of photosensitive, electrosensitive, or ionizable groups brings a key problem in their detection (Kanwar et al., 2017).

Moreover, BRs are present in very low amounts in plants, and complex MEs result in unreliable data and even quantitative error during mass spectrometry (Pan and Wang, 2009). Therefore, accurate qualitative and quantitative analysis of BRs is challenging. In this paper, a C_18_ cartridge SPE purification, DMAPBA-derived, online valve-switching system coupled with UHPLC-ESI-MS/MS was proposed for the quantification of trace BRs from plant samples. The method largely simplified the sample preparation procedure. In particular, the online valve-switching system reduced a large number of interfering substances from entering the mass spectrometer, such as residual DMAPBA and BRs-DMAPBA analogues, improved the stability and sensitivity of the mass spectrometer, and realized the accurate quantification of BRs in 100-mg of different organs of rapeseed. The developed online valve-switching system also has the potential to optimize the methods for the determination of analytes in other complex biological and environmental sample matrices.

The investigations of BR functions rely heavily on monitoring of the temporal and spatial variation of the BR concentrations (Symons and Reid, 2004; Fridman and Savaldi-Goldstein, 2013). Irani et al. (2012) have reported a fluorescent probe for BRs, which can visualize the probe labeled BR in plant tissues. In the method, chemical and genetic approaches are used to interfere with the trafficking of the BRASSINOSTEROID INSENSITIVE 1 (BRI1)–BRs complexes and examined their effect on BR signaling. However, the bioactive fluorescent BR analogues need to be synthesized artificially, and the result is an attenuation of the BR signal, rather than the exact level of BRs in plant. In addition, Tarkowská et al. (2016) have developed a sensitive mass spectrometry-based method that can simultaneously analyze 22 naturally occurring BRs in 50 mg of plant tissue extract without derivatization, and the samples they measured were rapeseed flowers, which had dozens of times higher BRs than the leaves or the roots. It remained unclear if their method was fit for the detection of samples with a low level of BRs. In this study, we have determined the precise contents of epiBL, epiCS, and d-epiCS in different tissues by UHPLC-ESI-MS/MS. The results indicated that the contents of epiBL, epiCS, and d-epiCS were all the highest in the flowers, and then in tender pods, and epiBL and epiCS gradually decreased from top to bottom with trunk (**Figures 7A–C**). These results are in accordance with the previous reports (Bajguz and Tretyn, 2003). Furthermore, the diagrammatic map outlined the spatial distribution of three BRs in rapeseed according to the data detected by UHPLC-ESI-MS/MS (**Figure 7D**). The spatial distribution of epiBL, epiCS, and d-epiCS in rapeseed tissues is helpful to accurately understand the signaling pathway and regulatory mechanism of BRs, and will facilitate the research of biosynthesis, accumulation, and transport mechanism for BRs (Gudesblat and Russinova, 2011). It is also helpful for the development of crop science and green agriculture.

## 5 Conclusion

We developed a C_18_ cartridge SPE purification, DMAPBA derivatization, and online valve-switching system coupled with the UHPLC-ESI-MS/MS method for detecting the contents of BRs in plant tissues. On this basis, we have successfully determined the contents of three structural types of BRs in various organ tissues of rapeseed, and outlined the spatial distribution map of BRs in plant. We can understand the spatial distribution of BRs in plants at the visual level for the first time. Furthermore, the online valve-switching system developed in this study also has the potential to determine various analytes in other complex biological and environmental sample matrices.

## Data availability statement

The original contributions presented in the study are included in the article/**Supplementary Material**. Further inquiries can be directed to the corresponding author.

## Author contributions

JT: Formal analysis, Validation, Writing – original draft, Writing – review & editing. WZ: Data curation, Formal analysis, Investigation, Writing – review & editing. KW: Data curation, Formal analysis, Investigation, Writing – review & editing. DD: Data curation, Formal analysis, Methodology, Validation, Writing – review & editing. LX: Funding acquisition, Project administration, Validation, Writing – review & editing.
