# Supplementary material for: Organ-level distribution tandem mass spectrometry analysis of three structural types of brassinosteroids in rapeseed
Source: Front Plant Sci. 2024 Mar 7;15:1308781. doi: 10.3389/fpls.2024.1308781 (PMC10956354; doi:10.3389/fpls.2024.1308781)
Supplement: Supplementary file 1 [file DataSheet_1.docx]

**[Supplementary material](https://www.overleaf.com/latex/templates/pnas-template-for-supplementary-information/wqfsfqwyjtsd#.WrAIzcPwZEZ)**

**For**

**Organ-level distribution tandem mass spectrometry analysis of three structural types of** **brassinosteroids in rapeseed**

**Jianhua Tong ^1^,** **Wenkui Zhao ^2^, Keming Wang ^3^,** **Danyi Deng ^1^, Langtao Xiao ^1^ ***

^1^ Hunan Provincial Key Laboratory of Phytohormones and Growth Development, Laboratory of Yuelu Mountain, College of Bioscience and Biotechnology, Hunan Agricultural University, Changsha 410128, China

^2^ College of Chemistry and Materials, Hunan Agricultural University, Changsha 410128, China

^3^ Assets and Laboratory Management Department, Hunan Agricultural University, Changsha 410128, China

ORCID IDs: 0000-0001-9785-9652 (J.T.); 0000-0003-1786-1950 (L.X.).

*Corresponding author: Langtao Xiao

E-mail: [ltxiao@hunau.edu.cn](mailto:ltxiao@hunau.edu.cn)

**
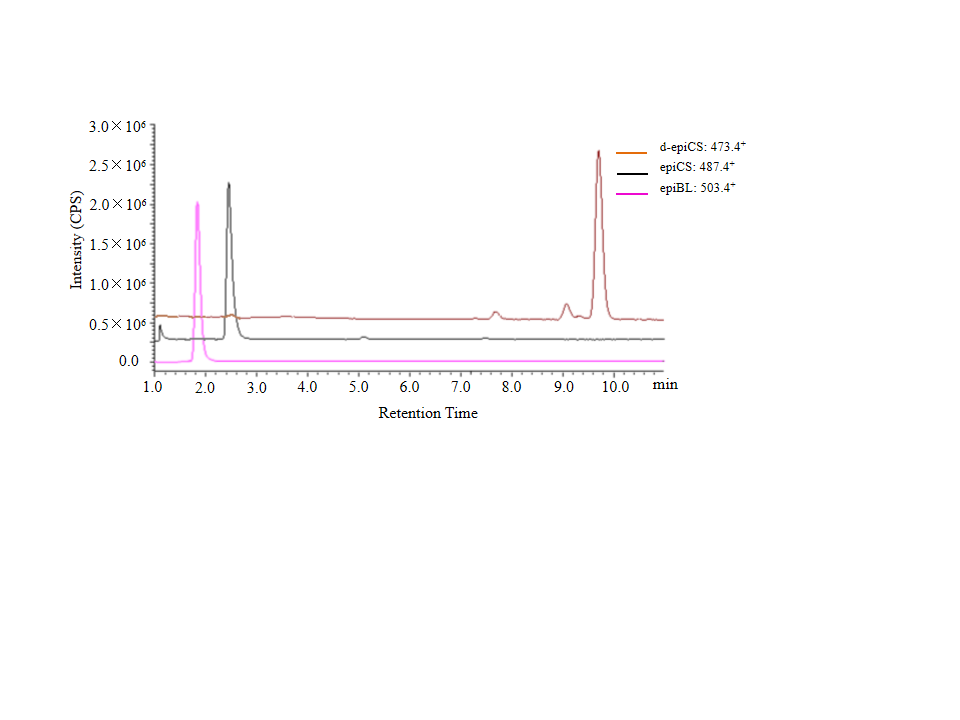
**

**Supplementary Figure 1** Selective ion chromatogram (SIC, M+Na^+^) of three BRs in SIM mode.

**
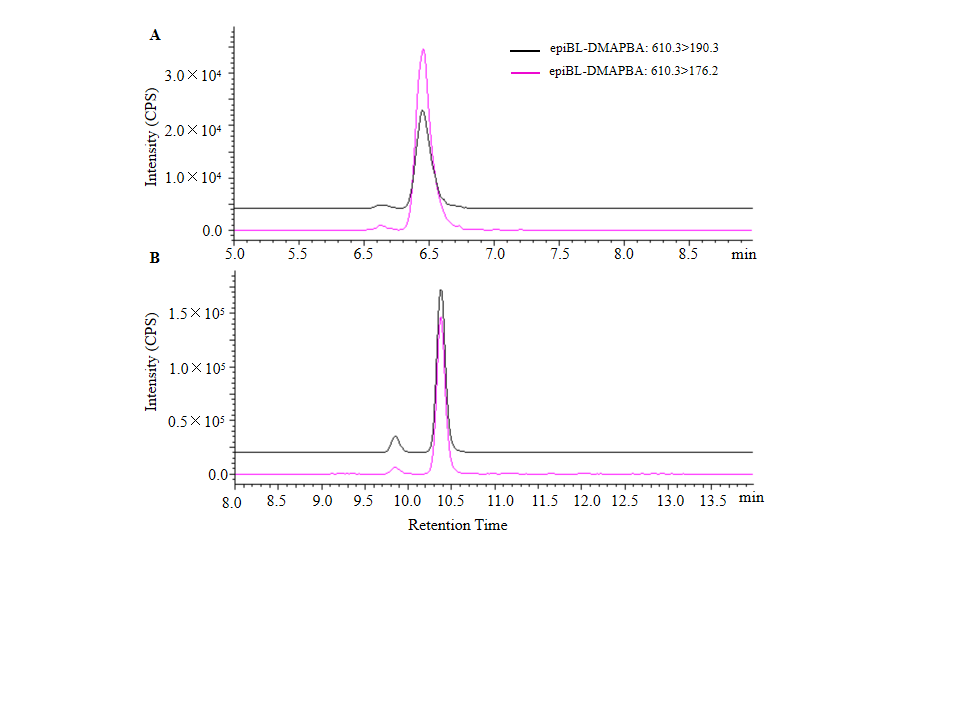
**

**Supplementary Figure 2** The EIC of epiBL-DMAPBA obtained with different mobile phase. **(A)** Acetonitrile/water system. **(B)** Methanol/water system.


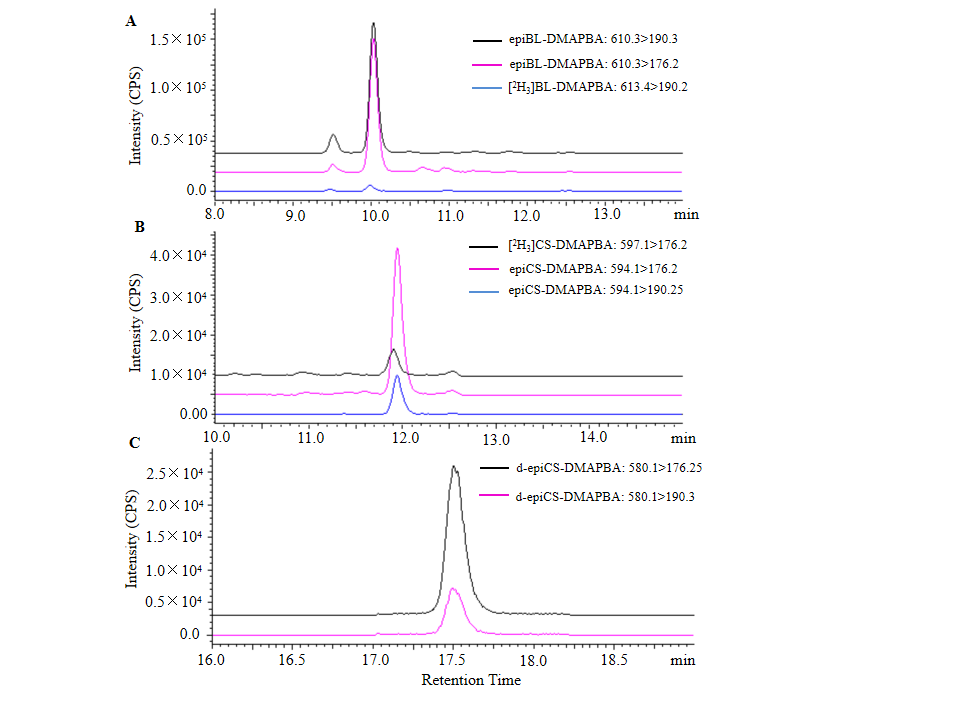


**Supplementary Figure 3** The EIC of epiBL-DMAPBA and [^2^H_3_]BL-DMAPBA (A), epiCS-DMAPBA and [^2^H_3_]CS-DMAPBA (B), d-epiCS-DMAPBA (C) of rapeseed tissue samples (100 mg FW tissues for each 50 μL sample) by online valve-switching-UHPLC-ESI-MS/MS.

**Supplementary Table 1** Comparison of analytical performance of our developed method and previously established methods (n.d.: not detected).

| Pretreatment  Method | Technical difficulties | The content of BRs detected (ng.g^-1^FW) | The amount of  plant tissues | Internal standard | Reference |
| --- | --- | --- | --- | --- | --- |
| MAX-MCX SPE purification and DMAPBA derivatization | Tedious, time-consuming and solvent-consuming of MAX-MCX SPE purification procedure | BL:0.01–2,  CS: 0.1–5,  TY: 0.1–5,  TE: n.d.–0.9 | 1 g samples of rice and *Arabidopsis* | [^2^H_3_]-BL, [^2^H_3_]-CS | [Xin](https://xueshu.baidu.com/s?wd=author:(Peiyong%20Xin)%20&tn=SE_baiduxueshu_c1gjeupa&ie=utf-8&sc_f_para=sc_hilight=person) et al., 2013 |
| Tandem SPE  purification-centriconfilter and BPBA derivatization | Tedious, time-consuming and solvent-consuming of tandem SPE purification procedure | 24-epiBR: 0.55,  28-epihomoBR: 0.07,  28-homoBR: n.d. | 2 g *Arabidopsis* leaves | Not used | Huo et al., 2012 |
| MSPE purification and DMAPBA derivatization | Synthesis of magnetic solid phase extraction cartridges (TiO_2_/MHMSS) | CS: 0.09–0.26,  BL: 0.04–0.13,   1. norBL/28-norCS/   28-homoBL : n.d. | 100 mg leaf and shoot of rice and rape | [^2^H_3_]BL,  [^2^H_3_]CS | Ding et al., 2014 |
| PT-SPE purification and BTBA derivatization | Preparation of PT-SPE cartridges and  synthesis of BTBA | 24-epiBL: 0.16–2.09,  24-epiCS: 0.045–0.29,  TY: 0.21–0.34,   1. d-epiCS/TE: n.d. | 5 mg leaf of rice seedling | Not used | Deng et al., 2016 |
| Polymer monolith microextraction and in-situ derivatization (PMME-ISD) | Preparation and characterization of monolithic column and installation of online PMME-ISD system | TY: n.d.–1.91  28-norCS:n.d.–0.88  CS:0.62–2.33  BL/ 28-norBL/  28-homoBL: n.d. | 1.0 mg tissues of rice, *Phaseolus vulgaris* L. flower or *Vigna unguiculata* flower; 1.3-mg *Arabidopsis* flower | [^2^H_3_]TY,  [^2^H_3_]CS, [^2^H_3_]BL [^2^H_3_]28-norBL | Wang et al., 2020 |
| 2DμSPE purificationm-APBA derivatization | Preparation of on-line 2DμSPE column and installation of 2DμSPE-OCD system | 24-epiBL: n.d.–0.11,  CS: 0.3–0.72,  d-epiCS: 0.3–4.5,  TE: 0.034–0.19,  TY: 0.04–0.15 | 225 mg tomato  leaves | Not used | Wu et al., 2013 |
| RhB-BA derivatization | Synthesis of RhB-BA | BL: 3.06–247,  CS: 4.02–186,  TY: 0.34–12.9,  28-norBL: n.d.–37.2,  28-norCS: n.d.–134,  28-homoBL: n.d. | 10 mg organs of rape flower | [^2^H_3_]BL,  [^2^H_3_]28-norBL,[^2^H_3_]CS, [^2^H_3_]TY | An et al., 2020 |
| MCX@BBII  purification and in situ derivatization | Synthesis of BBII; preparation  of MCX@BBII | BL: n.d.–157.28,  CS: n.d.–73.79,  TY: n.d.–4.03,  28-homoBL: n.d.–3.54,  28-homoCS: n.d.–3.55,  6-deoxoCS: n.d.–39.26 | 10–20 mg rice or *Arabidopsis*,  1 rape stamen | [^2^H_3_]BL,  ^[2^H_3_]CS,  [^2^H_3_]TY | Luo et al., 2018 |
| in-line coupled MSPD-MAX-MCX SPE purification and m-APBA derivatization | Tedious,time-consuming and solvent-consuming of MSPD-MAX-MCX SPE purification procedure | 24-epiBL: n.d., 5.26  24-epiCS: 0.12, 0.027  d-epiCS: 22.43, 0.19  DS: 1.68, n.d.  TE:0.10, 0.05  TY:2.17, 0.41 | 0.05 g of rice at booting stage and 0.2 g of rice at maturity stage | Not used | Wang et al., 2014 |
| C_18_ cartridge SPE purification, DMAPBA derivatization and online valve-switching system | Installation of online valve-switching system | epiBL:0.51–174.00,  epiCS: 0.22–28.62,  d-epiCS:11.79–475.11 | 100 mg different rapeseed organs | [^2^H_3_]BL, [^2^H_3_]CS | This work |
